# Identifying Loci Influencing 1,000-Kernel Weight in Wheat by Microsatellite Screening for Evidence of Selection during Breeding

**DOI:** 10.1371/journal.pone.0029432

**Published:** 2012-02-06

**Authors:** Lanfen Wang, Hongmei Ge, Chenyang Hao, Yushen Dong, Xueyong Zhang

**Affiliations:** Key Laboratory of Crop Germplasm Resources and Utilization, Ministry of Agriculture, The National Key Facility for Crop Gene Resources and Genetic Improvement, Institute of Crop Science, Chinese Academy of Agricultural Sciences, Beijing, China; University of Illinois, United States of America

## Abstract

Chinese wheat mini core collection (262 accessions) was genotyped at 531 microsatellite loci representing a mean marker density of 5.1 cM. One-thousand-kernel weights (TKW) of lines were measured in five trials (three environments in four growing seasons). Structure analysis based on 42 unlinked SSR loci indicated that the materials formed two sub-populations, viz., landraces and modern varieties. A large difference in TKW (7.08 g, *P<0.001*) was found between the two sub-groups. Therefore, TKW is a major yield component that was improved in the past 6 decades; it increased from a mean 31.5 g in the 1940s to 44.64 g in the 2000s, representing a 2.19 g increase in each decade. Analyses based on a mixed linear model (MLM), population structure (Q) and relative kinship (K) revealed 22 SSR loci that were significantly associated with mean TKW (MTKW) of the five trials estimated by the best linear unbiased predictor (BLUP) method. They were mainly distributed on chromosomes of homoeologous groups 1, 2, 3, 5 and 7. Six loci, *cfa2234-3A*, *gwm156*-3B, *barc*56-5A, *gwm234*-5B, *wmc*17-7A and *cfa2257*-7A individually explained more than 11.84% of the total phenotypic variation. Favored alleles for breeding at the 22 loci were inferred according to their estimated effects on MTKW based on mean difference of varieties grouped by genotypes. Statistical simulation showed that these favored alleles have additive genetic effects. Frequency changes of alleles at loci associated with TKW are much more dramatic than those at neutral loci between the sub-groups. The numbers of favored alleles in modern varieties indicate there is still considerable genetic potential for their use as markers for genome selection of TKW in wheat breeding. Alleles that can be used globally to increase TKW were inferred according to their distribution by latitude and frequency of changes between landraces and the modern varieties.

## Introduction

China is the largest wheat producer and consumer in the world, with 23.6 million ha, a mean 4,762 kg/ha, and a 112 million tonnes total production in 2008. There is long history of wheat cultivation in China extending over more than 2,000 years. Production extends from latitude 22°49′ to 48°03′ and much progress has been achieved in breeding and production in the last 60 years. Average wheat yields increased annually by 1.9% and production increased more than six-fold [1]. Thousand-kernel weight (TKW), as one of three major components of yield in wheat, has steadily increased over the period. Based on phenotyping of 1,800 cultivars released since the 1940s, TKW increased from a mean 31.5 g in the 1940s to 44.64 g in the 2000s, with a 2.19 g increase in each decade (Zhang et al. unpublished). Previous studies also showed that TKW was one of the three yield components with highest heritability, which varied from 59% to 80% [2]. Most genes affecting TKW have additive effects. Selection for TKW in the early generations of breeding is highly effective [2].

Crop domestication is an artificial evolutionary process of combining traits to meet human needs. During the domestication of cereals, for example, reductions in plant height to avoid lodging, large spikes, increased grain size, and disease resistance, were selected and conserved. Modern breeding involved further directional selection, which resulted in lower genetic diversity within the domesticated population than in the entire species. At the genome level, only a small number of genes (alleles) were positively selected and conserved [3]. Many other alleles at specific loci were gradually eliminated, leading to reduced genetic diversity at these loci compared with those present in the entire species. Diversity in genomic regions flanking the target genes was simultaneously reduced because of linkage. This phenomenon is referred to as linkage drag, hitchhiking, or selection sweep [4]. Hitchhiking generally leads to reduced diversity at target loci, linkage disequilibrium at loci surrounding the selected gene, and changed distribution patterns of alleles within the selected region [5], [6]. These effects also provide the bases for association of neutral markers, such as SSR and DArT, with agronomic traits [6]–[10].

We established a Chinese common wheat core collection (CC) and a mini core collection (MCC) after genotyping 5,029 candidate accessions at 78 SSR loci [11]. Choice of candidate entries was based on documentary data in the national gene bank [12]. The MCC contains 231 accessions, or 1% of the basic collection (23,135 accessions) with an estimated 70% representation of the genetic variation in that collection [11], [13]. The higher genetic diversity and artificial diminishment of dominant allelic frequencies in the MCC makes it a suitable population for detection of major QTLs controlling yield traits. It was shown to be a good reference set for revealing geographic distribution and time changes of important functional genes [10], [14]–[18]. In this study, we target loci associated with TKW to show the value of the MCC in dissecting complex yield traits in wheat. This association analysis provides useful information for marker-assisted selection in breeding wheat for increasing yield.

## Results

### Phenotypic Assessment

TKWs of the Chinese mini core wheat collection were measured in 4 growing seasons and 3 environments, including Luoyang, Henan province 2002, 2005, and 2006; Shunyi, Beijing 2010; and Qingdao, Shandong 2010 (Table 1). Minor differences in mean TKW occurred among different planting environments, and there were major differences between landraces and modern varieties. The MTKW of modern varieties (39.23 g) calculated using BLUP methods based on multiple environments was significantly higher (*P<0.001*) than that of landraces (32.15 g), confirming that TKW was a yield trait improved by breeding. The maximum TKW was not in a modern variety, but was in a landrace. This indicates that further genes for this trait are present in landraces and can be accessed for breeding. The total agronomic data were considered in whole genome association analysis.

**Table 1:** Comparison of 1,000-kernel weights between landraces and modern varieties in the Chinese wheat mini core collection in the 5 environments.

		TKW-L02	TKW-L05	TKW-L06	TKW-S10	TKW-Q10	MTKW
Mean	Landraces	33.72±0.58	30.66±0.51	33.06±0.43	32.23±0.47	30.37±0.51	32.15±0.37
	M varieties	41.69±0.74	38.67±0.67	40.81±0.65	39.67±0.73	37.87±0.64	39.23±0.54
p-value		3.59E-15	3.98E-18	1.03E-20	1.51E-16	5.45E-17	1.33E-23
Min	Landraces	23.78	16.47	22.65	20.22	17.06	22.78
	M modern varieties	23.5	23.46	27.7	22.9	23.76	26.56
Max	Landraces	57.56	53.94	53.66	55.02	54.48	52.57
	M varieties	55.68	49.91	53.43	53.58	52.12	49.99

L: Luoyang, Henan province; S: Shunyi, Beijing; Q: Qingdao, Shandong province.

### Population Structure Analysis

**Figure 1:**
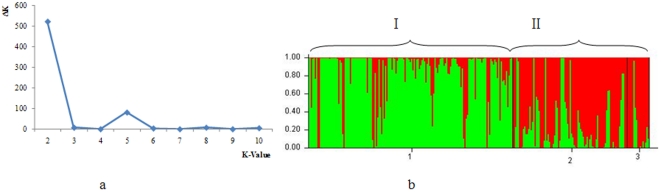
Population structure analysis of 262 wheat cultivars based on 42 unlinked SSR loci. a: Population structure as determined by Structure v2.2 analysis. Since Δk peaks at k = 2, the varietal set was split into two sub-groups. b: Structure analysis reveals that the 262 wheat cultivars are clustered into two sub-populations. I. Landrances. II. Modern varieties and introduced lines.

Population structure analysis can identify locus associations that are statistically significant, but biologically invalid due to strong correlation with population structure. However, if the population structure is properly dealt with, the likelihood of spurious associations can be minimised [7], [19]. Forty-two loci distributed across every arm of the 21 wheat chromosomes were chosen to examine the population structure of entries in the mini core collection. We selected K values of assumed groups from 1 to 10. After 80 cycles of simulation, we found that K = 2 was the best separator providing the highest delta k value, and showing that the MCC entries comprised two sub-populations. One group was mainly the landraces, and the other included modern varieties and introduced lines (Fig. 1). Overlapping occurs between the two groups because in the early breeding period (1940–1960s), most of the released varieties were derived from crosses between Chinese landraces and introduced European or American varieties [20]. This was consistent with results based on 512 SSR loci using a similar set of materials [13].

### SSR Loci Associated with TKW

**Figure 2:**
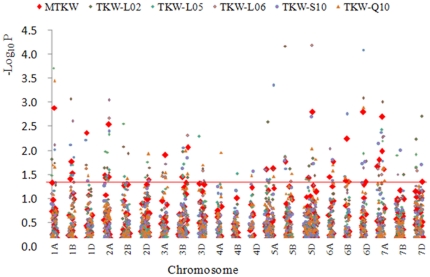
Genome wide association analysis of 1,000-kernel weight with SSR loci. TKWs collected from 5 trials were used to estimate mean values (MTKW). TKW-L02, TKW-L05, TKW-L06, TKW-S10, and TKW-Q10 indicate 1,000-kernel weights in 2002, 2005 and 2006 in Luoyang (Henan province), 2010 in Shunyi (Beijing) and Qingdao (Shandong), respectively.

We firstly used the MLM model [21] to make a marker/MTKW (TKW) association analysis. Thirty-two loci were significantly (*P<0.05*) associated with MTKW. An association with *cfa2257* on 7AL was detected in all five trials; 8 loci were detected in four trials, i.e. *wmc304-1A*, *wmc147-1D*, *gwm312-2A*, *gwm547-3B*, *gwm234*-*5B*, *gwm174*-*5D*, gwm55-*6D*, and *wmc17-7A*; 6 loci were detected in three environments, viz., *gwm268-1B*, *cfa2234-3A*, *gwm156-3B*, *cfd266-5D*, *gwm356-6A* and *gwm471-7A*; 9 loci in two environments, and eight loci were detected in one trial (Fig. 2, Table S1).

Among the 24 loci associated with MTKW in at least two trials, we found breeder-favored alleles with strong positive effects on MTKW at 22 loci; and they were mapped to 11 chromosomes, viz. 1A, 1B, 1D, 2A, 3A, 3B, 5A, 5B, 5D, 6D, and 7A. The 7A effect spanned four loci, including *gwm471*, *wmc168*, *wmc17* and *cfa2257*. The genetic distance between *wmc17* and *cfa2257* is 2.72 cM. No stronger linkage disequilibrium (LD) was found between the two loci (r*^2^* = 0.10, P>0.05) indicating they may not relate to a single yield gene, a result also suggested by previous QTL studies of TKW [22]–[26]. Three loci on chromosomes 1B and 2A were associated with MTKW. The allelic effect at each locus on MTKW was estimated by ANOVA (SPSS16). Significant or extremely significant differences in MTKW were detected between varieties with the favored allele and those with other alleles. Six loci with the strongest effects, and individually explaining more than 10% of the total variation were detected on chromosomes 3A, 3B, 5A, 5B and 7A (*R^2^*>10%) (Table 2).

**Table 2:** Favored alleles, their frequencies, genetic effects and *R^2^* at 22 SSR loci significantly (*P<0.05*) associated with MTKW.

Loci	Chr.	Genetic Position (cM)	Favored allele (bp)	Freq. (%)	MTGW (mean±S.E)	*P* value	*R^2^* (%)	Times associated	QTL reported [Reference No.]
*cfa2153*	1A	15.4	198	28.24	36.58±0.67	0.002**	3.43	2	
			others	71.76	34.09±0.41				
*wmc304*	1A	52.0	126	16.41	37.83±0.87	1.31E-4***	5.12	4	[34]
			others	83.59	34.19±0.38				
*gwm11*	1B	57.0	199	15.27	37.08±0.89	0.006**	2.47	2	[25], [34], [36]
			others	84.73	34.38±0.38				
*gwm403*	1B	61.4	134	22.14	36.29±0.81	0.024*	1.56	2	[25], [65]
			others	77.86	34.36±0.39				
*gwm268*	1B	75.2	230	8.02	39.80±1.45	2.58E-5***	6.23	3	
			others	91.98	34.35±0.35				
*wmc147*	1D	0	150	22.14	37.31±0.81	1.41-4***	5.07	4	[35]
			others	77.86	34.08±0.38				
*gwm275*	2A	56.12	110	11.45	38.87±0.85	3.02E-5***	6.13	2	[26], [34], [38]
			others	88.55	34.26±0.37				
*gwm312*	2A	79.26	190	16.41	38.55±0.91	1.98E-6***	7.99	4	[22], [26]
			others	83.59	34.05±0.37				
*gwm372*	2A	80.45	331	6.11	40.93±1.80	8.16E-6***	7.03	2	[22], [26]
			others	93.89	34.39±0.35				
*cfa2234*	3A	107.38	142	43.89	37.59±0.49	5.00E-13***	18.2	3	[34]
			others	56.11	32.6±0.43				
*gwm156*	3B	40.05	311	11.45	40.91±1.07	1.56E-10***	14.27	3	[34]
			others	88.55	34.00±0.35				
*gwm547*	3B	100.0	null	11.45	39.54±0.83	1.00E-6***	8.46	4	[34]
			others	88.55	34.18±0.37				
*barc56*	5A	18.8	119	27.86	38.02±0.60	6.46E-9***	11.84	2	[24], [25], [34], [36]
			others	72.14	33.54±0.40				
*gwm234*	5B	20.57	237 and 239	16.79	40.05±0.83	4.86E-12***	16.48	4	[26]
			others	83.21	33.73±0.35				
*wmc415*	5B	61.29	154	40.46	36.38±0.58	2.85E-4***	4.84	2	[37]
			others	59.54	33.71±0.43				
*cfd266*	5D	22.0	167	19.08	37.49±0.82	1.99E-4***	4.83	3	
			others	80.92	34.15±0.38				
*gwm174*	5D	51.85	191	9.92	40.01±1.13	6.86E-7***	8.71	4	[65]
			others	90.08	34.22±0.36				
*gwm55*	6D	83.45	130	21.76	37.82±0.74	5.09E-6***	7.35	4	[36]
			others	78.24	33.95±0.39				
*gwm471*	7A	1.0	109	6.11	41.14±1.39	3.85E-6***	7.54	3	[23], [34]
			others	93.89	34.38±0.35				
*wmc168*	7A	32.9	307	8.4	37.93±1.50	0.007**	2.35	2	[23], [34]
			others	91.6	34.5±0.36				
*wmc17*	7A	89.17	182 and 184	37.4	38.12±0.55	3.09E-14***	19.62	4	[23], [24]
			others	62.6	32.80±0.39				
*cfa2257*	7A	91.89	129	20.61	40.13±0.71	5.99E-16***	21.99	5	[22], [25], [26]
			others	79.39	33.41±0.35				

*: P<0.05;

**: P<0.01;

***: P<0.001.

*R^2^*: indicates the percentage of total variation explained.

Genetic positions of SSR markers on chromosome 1A to 7D were based on Röder et al. [54]; Somers et al. [55] and http://www.shigen.nig.ac.jp/wheat/komugi/maps/markerMap.jsp.

Two favored alleles were detected at *gwm234 and wmc*17, respectively. To simplify the data process, the two alleles were considered to be the same in estimating their genetic effects on MTKW.

### The Distribution of Favored Alleles at Associated Loci

**Figure 3:**
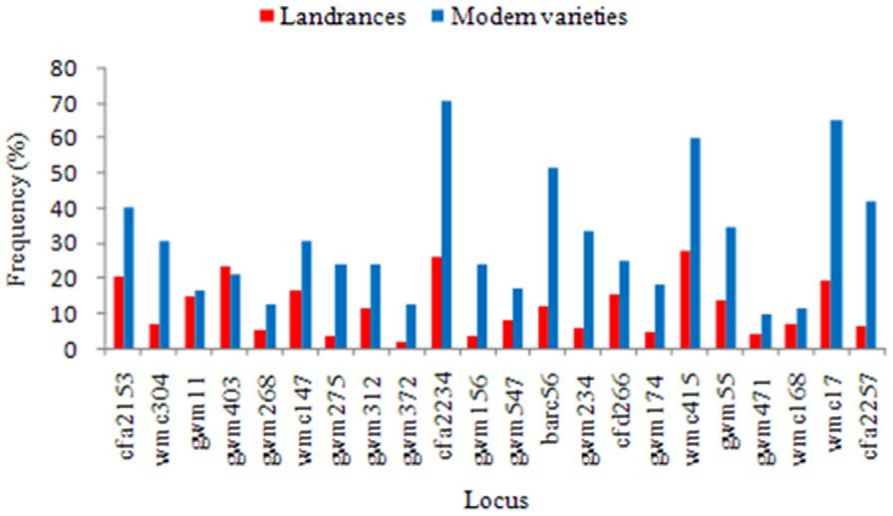
Comparative frequencies of favorable alleles at 22 loci for landraces and modern varieties in the Chinese wheat mini core collection.

We estimated the frequencies of favored alleles at each of the 22 loci in the landrace and modern entries groups in the mini core collection. Except at *gwm403-1B*, favored allele frequencies were much higher in modern varieties than in the landraces (Figure 3, Table S2). This reflects positive selection of those alleles in breeding programs.

Modern varieties usually have fewer allelic variations than landraces [13]. However, the major allele frequency is not always higher in modern varieties than in landraces (Table S3). At the 42 loci without obvious signs of selection, the average major allelic frequency increased from 28.46% in landraces to 32.87% in modern varieties, and the *t*-test indicated that the change was not significant (*t* = 1.661, *p* = 0.1), but with equal variances (*F* = 1.23)<*F_0.05_* = 1.69). However, at the 22 loci associated significantly with MTKW, their average frequency increased from 11.58% to 30.52%, an extremely significant difference (Table S2; *t* = 4.591, *p* = 7.95E-05), and unequal variances (*F* = 5.13, *F_0.05_* = 2.07).

Among the four loci with favored allelic frequencies higher than 50% in modern varieties, *cfa2234*-*3A*, *barc56-5A* and *wmc17-7A* were among the six loci with the highest effects on phenotype variation of TKW (Table 2). In addition, dramatic increases were also detected at *wmc17* and *cfa2257* on 7A (Table S2); these were also among the six loci (Table 2). The increased numbers and frequencies of favored alleles were accompanied by increased mean MTKW in modern varieties (Table 3). Therefore, we believe that the increase in favored allele frequencies at the 22 loci was mainly caused by selection for grain size over the five decades before 2000 (Table S2).

**Table 3:** Number, frequency and mean MTKW of landraces and modern varieties in the mine core collections.

No. of favored alleles		0	1	2	3	4	5	6	7	8	9	10	11	12	13	15
Number	Landrances	15	36	43	25	15	10	6	2	3	1	1				
	Modern varieties	2	1	1	6	3	17	13	10	11	11	4	2	3	3	1
Freq (%)	Landrances	9.55	22.93	27.39	15.92	9.55	6.37	3.82	1.27	1.91	0.64	0.64				
	Modern varieties	2.27	1.14	1.14	6.82	3.41	19.32	14.77	11.36	12.5	12.5	4.55	2.27	3.41	3.41	1.14
MTKW±SE	Landrances	29.35±0.83	30.81±0.63	30.86±0.59	32.86±0.72	32.54±0.82	34.91±1.47	35.88±1.53	39.14±0.48	47.10±2.78	38.32±0	38.84±0				
	Modern varieties	27.56±1.01	31.45±.0	37.81±.0	35.67±1.23	34.45±1.84	35.94±0.82	38.79±1.17	39.52±0.99	40.93±1.16	43.34±0.72	42.81±3.50	45.70±4.29	42.80±2.42	45.90±0.53	44.01±. 0

Several columns with zero SE because there is only one cultivar in the group.

### Accumulation of Favored Alleles from Breeding

**Figure 4:**
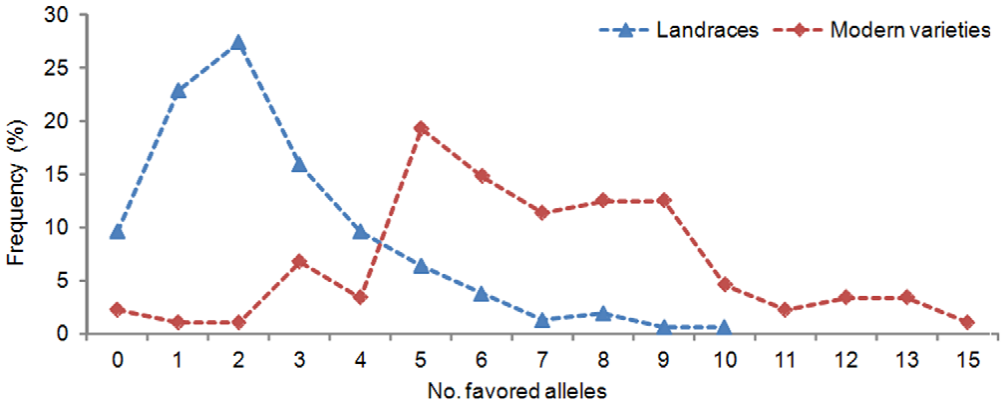
Accumulation of favorable alleles in landraces and modern varieties from different regions of China. Modern breeding promoted the accumulation of favored alleles.

Positive selection of favored alleles at key loci was also clearly implicated by changes in their number and frequency (Table 3). The best modern variety (44.01 g) had 15 favored alleles at 22 critical marker loci, whereas the best landrace (38.84 g) had 10. Almost 92% of the landraces had 0–5 favored alleles, whereas 85.2% of modern varieties had more than 5 favored alleles, ranging from 5–15. Modern breeding has significantly promoted the accumulation of favored alleles in varieties (Fig. 4). These results illustrate the reliability of identifying favored alleles. Importantly, no modern cultivar has favored alleles at all 22 marker loci (Table 3, Fig. 4), indicating further capacity for improvement of TKW by maker-assisted selection.

### Geographic Distribution of Favored Alleles at the Six Loci with the Highest Contributions to TKW

**Figure 5:**
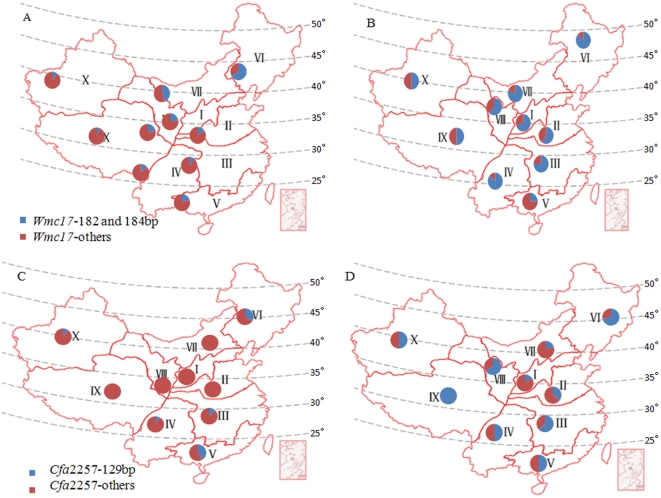
Favored alleles and their frequencies at the *cfa2257* and *wmc17* loci on chromosome 7AL in the Chinese wheat mini core collection in ten ecological regions in China. A and B indicate *wmc17* frequencies in landraces and modern varieties, respectively; C and D indicate *cfa2257* frequencies in landraces and modern varieties, respectively. Zone I: North winter region Zone II: Yellow and Huai River valleys, winter wheat region. Zone III: Middle and Low Yangtze River valleys, winter wheat region. Zone IV: Southwestern winter wheat region. Zone V: Southern winter wheat region. Zone VI: Northeastern spring wheat region. Zone VII: Northern spring wheat region. Zone VIII: Northwestern spring wheat region. Zone IX: Qinghai-Tibetan Plateau, spring-winter wheat region. Zone X: Xinjiang winter-spring wheat region. Source: Zhuang QS [20].

Closely located loci *cfa2257* and *wmc17* on chromosome 7AL with the highest contributions to TKW were chosen to analyze their distributions in different production regions in China (Figure 5). The favored alleles (182 bp and 184 bp) of *wmc17* occurred in both landraces and modern varieties, but their frequencies were significantly higher in modern varieties than in landraces. Among landraces the highest frequency of the favored allele with high TKW was in region VI with region VII in second place. Both of the regions grow spring wheats with high TKW. For modern varieties, regions IV and VI had the highest frequency, with VII in third place. Other regions showed large variations in the frequencies of favored alleles. Regarding *cfa2257*, the highest frequency of the favored 129 bp allele was in region V with region VI in second place, a little lower than its frequency in landraces in region V. This allele was not present in landraces from 5 wheat regions (I, II, VII, VIII, and IX), a situation clearly different from the modern variety group where all modern lines, for example in region IX, carried the favored allele. This allele was also common in varieties from regions VI and VIII and occurred in the other regions. The geographic distributions of favored alleles at four other loci are included in Figure S1.

### Genetically Additive Effects of Favored Alleles on TKW

**Figure 6:**
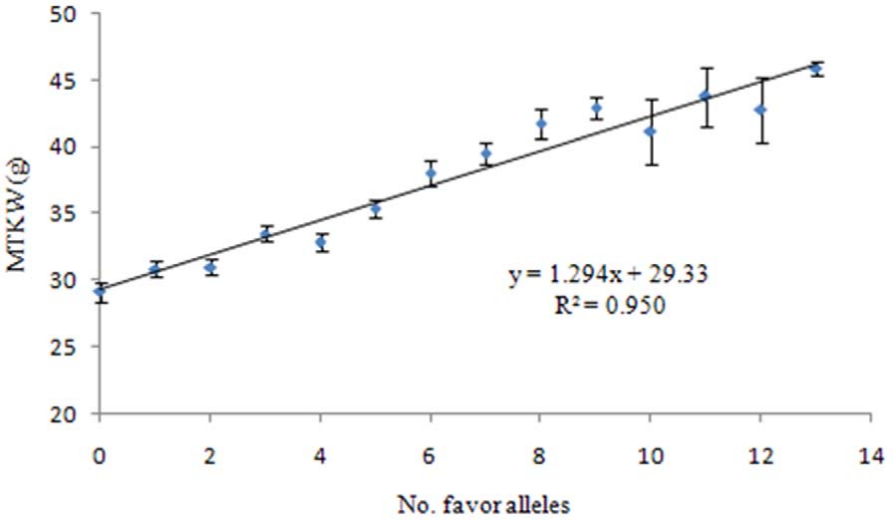
Linear regression analysis of MTKW based on five trials.

To determine if additive effects occur among the favored alleles at the 22 loci, we estimated the mean TKW of varieties with different numbers of favored alleles. There was a high linear correlation (Y = 1.294X+29.33, R^2^ = 0.95) between MTKW and number of favored alleles (Figure 6) indicating clearly additive effects of favored alleles. However, an obvious negative interaction among loci after the number of favored alleles reached 10 and resulting in larger differences between real and expected TKW cannot be ignored (Fig. 6). A confounding factor was that some subgroups included only one or two varieties (Table 3).

## Discussion

### SSR Loci Associated with TKW may Represent Major QTLs affecting Yield

According to Nordborg and Weigel [27], association mapping represents next-generation plant genetics. It uses ancestral gene associations and natural genetic diversity within a population to dissect quantitative traits, and is built upon the presence of linkage disequilibria. It offers a potentially powerful approach for mapping causal genes with modest effects [28], [29]. The association results and allelic effects are influenced by population type and size, and the breeding system of the species. Core collections are very suitable for association analysis of highly heritable and domestication traits [8]. In the Chinese wheat mini core collection, the mean LD decay distance for landraces at the whole genome level was <5 cM compared to 5–10 cM in modern varieties. Only 0.05% of marker pairs in significant (*P<0.001*) LD reached threshold levels of r*^2^* = 0.2 [13]. The observed LD is much lower than for CIMMYT historical breeding materials, but is similar to a population of European varieties released since the beginning of the last century [9], [30]. The overall population structure is very weak, but the two sub-populations, landraces and modern varieties, were clearly distinguished [11], [13]. This separation makes the MCC population suitable for marker/trait association analysis. Earlier analyses revealed differences in regard to latitude distribution and changes over time in important genetic haplotypes, such as those of *Pina* and *Pinb* [14], *Ppd-1* [15], *GS2* (glutamine synthetase) [17], *TaGW2* [18], *TaSus2* [10], [16]. However, compared with the candidate core entries, the frequencies of predominant alleles declined to enable the maximum representation of allelic variation at each locus [6], [11]. This likely reduced the association power, allowing the major QTLs to be targeted [8], [10], [29]. This was supported by the data in Table 2, i.e. most of the associated loci were detected within QTL intervals controlling TKW. Comparative analysis of modern varieties and landraces reveals major loci that have been almost fixed in modern varieties because of positive selection in breeding. For example, in wheat, two haplotypes coding an invertase gene on chromosome 5D were detected among 384 European wheat varieties released since the 1880s, with 382 being the same haplotype, and only two being the other. The latter would obviously have a very low chance of being detected in general association mapping populations. However, in our MCC, 58 accessions carried the above minority haplotype (Jiang YM and Zhang XY unpublished data).

### Integration of Association Mapping and QTL Mapping Generates More Reliable Results

Artificial selection (domestication and breeding) leaves strong foot-prints in plant genomes [4], [6], [10], [31]. Understanding the relationship between DNA sequence variation and variation in phenotypes for quantitative or complex traits will increase the speed of selection in breeding programs for predicting adaptive evolution [32]. Both linkage and association mapping aim to identify markers sufficiently closely linked to functional sequence variations (causal genes) encoding changes in phenotype, allowing breeders to select and manipulate these alleles routinely in diverse breeding populations [29].

Localization and interpretation of QTLs and associated loci provide confidence in results from association analysis [6], [27], [32]. In soybean,a high correlation (*R^2^* = 0.83) between the distribution of SSR markers and genes suggested close association of SSRs with genes [33]. This makes us believe that SSR markers are suitable for association analyses. Most of the associated markers were found in genomic regions where genes or quantitative trait loci (QTL) influencing the same traits were found previously. This provides an independent validation of the approach. Additionally, new chromosome regions for TKW were identified in the wheat genome through association analysis. Overall, 22 SSR loci on 11 chromosomes were associated with TKW with high confidence. This is much greater than the number of QTLs mapped in any bi-parental population, indicating the dissection power of this methodology in natural populations (Table 2) [34]–[36]. After genotyping 254 loci in 194 F_7_ recombinant inbred lines, Groos et al. [37] detected nine chromosome regions controlling TKW (chromosomes 1D, 2B, 2D, 3A, 5B, 6A, 6D, 7A, 7D). These are largely consistent with our association results (Table 2) from which three QTLs, on chromosomes 2B (*Xgwm148* - *Xgwm374* - *Xgwm388*), 5B (*Xgwm639* - *Xgwm271* - *Xgwm604*) and 7A (*Xcfa2049* - *Xbcd1930*) were detected in six environments. The QTL on 7A mapped to the middle to terminal region of 7AL, and partially overlapped the region *wmc17* - *cfa*2257 detected in the present study. QTL controlling TKW were also detected at a homologous region of 7DL [37]. Furthermore, the association mapping result for this region is much more precise than with QTL mapping; the genetic distance between the two nearest markers being only 2.72 cM (Table 2, http://www.shigen.nig.ac.jp/wheat/komugi/maps/markerMap.jsp). This raises the question of whether a single causal gene is involved. The *r^2^* value between the two markers is about 0.1 in the MCC. Thus there may be two linked causal genes, a possibility that is consistent with the obvious geographic distribution difference in favored alleles at two loci (Fig. 5). Similarly, *gwm312* and *gwm372* on chromosome 2A also reflect effects of two causal genes, which formed weak LD (r^2^ = 0.23) in the MCC population. These examples illustrate how haplotype and LD analyses enable dissection of yield QTLs in practice [10].

In another comprehensive QTL mapping report based on 12 data sets obtained over three years of trials with 2–5 environments/year, Snape et al. [38] detected seven relatively stable QTLs controlling TKW in 11 DH populations. These QTLs were distributed on chromosomes 2A (*gwm445*), 2B (*gwm148*), 2D (*wmc41*), 3A (*gwm428* - psp*3001*), 5A (*gwm293*), and 6A (*wmc32*, *gwm518*). The *gwm445*-associated QTL was not detected in the MCC population, but was detected in the core collection (1,160 entries) with a 2.89 g increase in TKW (Zhang and You unpublished); *gwm445* is very close to an almost orthologous region of chromosome 2D marked by *wmc41*. Both *gwm148* on 2B and *gwm275* on 2A mapped to orthologous regions detected in our study (Table 2). Loci *gwm55-6D* and *gwm415-6B* associated with TKW may be homologous to a QTL on 6A flanked by *wmc32* and *gwm518* in the pericentromeric region, in which *TaGW2* is located [18](http://wheat.pw.usda.gov/ggpages/SSRclub/GeneticPhysical/). In addition, distinct changes in frequencies of SSR alleles between the landraces and modern varieties at the 22 loci caused by hitchhiking effects provided positive evidence of selection for specifically favored alleles (Fig. 4; Table S2) [4], [6].

### Linear Correlation between TKW and Favored Alleles Showing the Practical Value of Genome Selection in Breeding

Compared with QTL mapping, another attribute of association analysis is the validation of favored alleles in germplasm collections [8]. For example, Röder et al. [39] mapped a major TKW QTL to the interval *Xgwm295* - *Xgwm1002* located in the distal telomeric bin (7DS4-0.61-1.00) in the physical map of wheat chromosome 7DS. Zhang et al. [6] found that allele *Xgwm130_132_* underwent very strong positive selection during modern breeding. *Xgwm130* maps between *Xgwm295* and *Xgwm1002*, with a genetic distance of 1.1 cM from *Xgwm295*. Thus the identification of favored alleles will help in choosing parents for crossing programs, to ensure maximum levels of favored alleles across sets of loci targeted for selection, and to promote fixation at these loci [40].

Whereas linear correlations between TKW and favored alleles indicate the additive effects of QTLs or genes, the possibility of other genetic effects should not be ignored in practice. Higher standard errors when the numbers of favored alleles exceed 10 (Figure 6) reveals the possibility of threshold effects with excessive numbers of favored alleles. Another cause of the higher standard errors was that the number of varieties carrying more than 10 favored alleles was much fewer (Fig. 4).

The concept of genome-wide selection (GWS) was recently introduced in plant breeding; this method uses information from all markers, as opposed to significant markers, to evaluate the breeding value of each line [41], [42]. Frisch et al. [43] used transcription data from 46,000 oligonucleotide arrays to develop a prediction model for the value of parental maize lines in relation to the grain yield performance of their hybrid progeny. They found that predictions based on 50 well chosen genes were as accurate as predictions based on 5,000 random genes. Therefore, the combination of GWA and GWS will in future enhance the practical application of GWS in crop improvement [29]. This work paves the way for further targeted diversity mining in landrace populations and wild relatives via comparative genomics analysis. The most interesting example is that genes on a *Thinopyrum ponticum* group 7L chromosome enhance grain yield by 13% in the genetic background of newly released varieties [44], [45]. The 7L gene may be orthologous to the TKW chromatin block flanked by *wmc17* and *cfa2257* on 7AL (Table 2) [46]. These examples indicate that increased grain weight in wheat is feasible using genomic selection.

### Frequency and Geographical Distribution of Favored Alleles Indicate Potential for Yield Increases by Selection of Loci Associated with TKW

In wheat, some genes or SSR loci associated with yield vary across latitudes, such as *TaSus2* on chromosome 2B [16], *TaGW2* on 6A [18] and *gpw7596* on 7B (EST-SSR) [47]. Favored alleles usually occur at relatively lower latitudes. This might indicate that the functional genes at these loci, including mapped alleles and those linked with markers, might be responsive to sunlight and temperature during the growing season [48], [49]. None of the 6 SSR loci with determination coefficients higher than 10% associated with favored MTKW alleles *cfa2234_142_* (3AL), *gwm156_311_* (3BS), *barc56_119_* (5AS), *wmc17_182_*, *_184_* (7AL) and *cfa2257_129_* (7AL) had obvious correlations with latitude (Fig. 5, Fig. S1). They can therefore be used globally for increasing TKW. None of the 88 genotyped modern varieties, and 17 introduced lines, carried favored alleles at all 22 loci, and only one variety had 15 favored alleles (Table 3, Fig. 4). Therefore, there are still opportunities for maker-assisted selection for TKW in wheat breeding.

## Materials and Methods

### Phenotypic Assessment

A Chinese wheat mini core collection [6], [11], [13] was chosen for genome-wide association of 1,000-kernel weight (TKW) using SSR markers. The mini MCC contained 262 wheat lines including 157 landraces, 88 modern varieties, and 17 introduced lines representing 1% of the national collection, but more than 70% of its genetic diversity [11]. The phenotype data were collected in five environments, viz. 2002, 2005 and 2006 in Luoyang, Henan province, and 2010 in both Shunyi, Beijing, and Qingdao, Shandong. The field planting design and methods of TKW measurement were described in Su et al. [18] and Jiang et al. [16]. Mean values of TKW and standard errors were analyzed by SPSS 16.0 (http://www.brothersoft.com/downloads/spss-16.html). The mixed mean TKW (MTKW) was estimated by the best linear unbiased predictor (BLUP) method according to Bernardo [50]–[52].

### SSR Genotyping

Genomic DNA was extracted from young leaves of 10 seedlings of each entry according to Sharp et al. [53] and fingerprinted by PCR amplifications that identified alleles at 531 SSR loci. Genetic map positions for most of the markers (512 loci) can be found in Hao et al. [13]. The loci were distributed evenly across all 21 wheat chromosomes. The primer sequences and genetic locations of the loci were obtained from http://www.shigen.nig.ac.jp and http://wheat.pw.usda.gov [54], [55]. The annealing temperature for each primer pair was obtained from Röder et al. [54] and GrainGenes (http://wheat.pw.usda.gov). After purification, the amplified PCR products were separated on an ABI3730 DNA Analyzer (Applied Biosystems, Foster City, CA, USA). Fragment sizes were determined using an internal size standard (LIZ500, ABI, USA), and the outputs were analyzed using GeneMapper software (http://www.appliedbiosystems.com.cn/). The minor allele frequency (MAF) was set as 0.05 during the following statistics.

### Association Analysis

To reduce the risk of false or spurious associations, population structure was estimated by STRUCTURE v2.2 software according to Pritchard and Rosenberg [56] and Pritchard et al. [57], based on 42 unlinked loci from both arms of each chromosome with a length of burn-in period equal to 50,000 iterations and a run of 500,000 replications of Markov Chain Monte Carlo (MCMC) after burn in. A total of 80 independent runs were set with the number of presumptive groups (k) varying from 1 to 10. In order to select the most appropriate number of sub-groups, the Δk value, based on the average Ln probe of each run, was calculated allowing the internal population structure of the sample set to be determined [58], then Q data were obtained according to the corresponding K value.

In order to define the degree of genetic covariance between pairs of individuals, a kinship (K) analysis was conducted by genotypic data with SPAGeDi software [59]. The calculation of pairwise kinship coefficients was according to Loiselle et al. [60] with 10,000 permutation tests. Negative values between individual pairs were then set to 0, as this indicated that they were less related than random individuals [21].

The mixed linear model (MLM) module with Q+K of the TASSEL 2.1 software package (http://www2.maizegenetics.net/) [61], [62] was used for genome wide association of MTKW and TKW in each trial. The relative value of the favored allele for TKW (*R^2^*) was calculated according to the equation, *R^2^* = (SSA−f_A_×MSE)/SST where SSA indicated the sum of squares between groups of favorable alleles and others, f_A_ indicated the degrees of freedom of the group with the favored alleles, MSE indicated the error mean square, and SST indicated the sum of squares [62], [63].

Because modern varieties usually have fewer alleles than the landraces generally, frequency at most alleles would be increased in modern varieties [13]. To avoid circular reasoning in data interpretation, we randomly selected one locus on each arm of the 21 chromosomes, with PIC values higher than the global mean (0.65), for evaluating changes in major allele frequencies between the two sub-populations at loci associated significantly with MTKW and loci probably not removed by selection in domestication and breeding [64] (Table S2, Table S3). We used *F*-tests and *t*-tests to estimate differences in allelic frequencies between the landrace and modern variety groups by SPSS15.0.

## Supporting Information

Figure S1 **Favored allele frequencies (in blue) in landraces (left) and modern varieties (right) at the** ***barc56, cfa2234, gwm156*** **and** ***gwm234*** **loci.**
(TIF)

Table S1 **SSR loci associated with MTKW and TKW in 5 environments by Tassel 2.1(P<0.05).**
(DOCX)

Table S2 **Frequency change of favored alleles at the 22 loci associated with MTKW in landraces and modern varieties.**
(DOCX)

Table S3 **Frequency change of major alleles at the 44 loci with higher** ***PIC*** **than the mean (0.54) in landraces and modern varieties.**
(DOCX)
