# Supplementary figures and images for: Identifying Loci Influencing 1,000-Kernel Weight in Wheat by Microsatellite Screening for Evidence of Selection during Breeding
Source: PLoS One. 2012 Feb 6;7(2):e29432. doi: 10.1371/journal.pone.0029432 (PMC3273457; doi:10.1371/journal.pone.0029432)

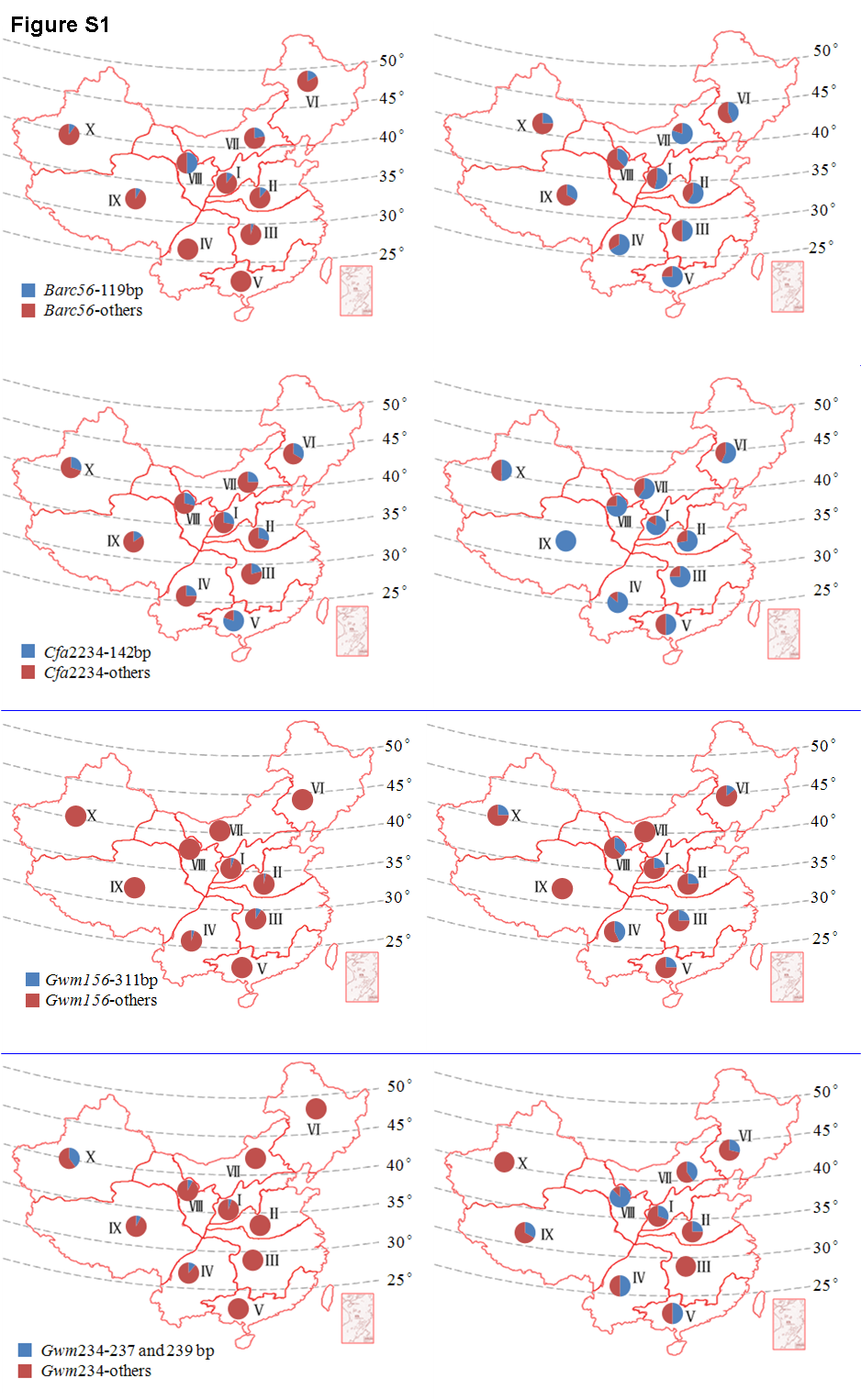

Supplement: Figure S1 — Favored allele frequencies (in blue) in landraces (left) and modern varieties (right) at the barc56, cfa2234, gwm156 and gwm234 loci. (TIF) [file pone.0029432.s001.tif]
