# Supplementary material for: Identifying Loci Influencing 1,000-Kernel Weight in Wheat by Microsatellite Screening for Evidence of Selection during Breeding
Source: PLoS One. 2012 Feb 6;7(2):e29432. doi: 10.1371/journal.pone.0029432 (PMC3273457; doi:10.1371/journal.pone.0029432)
Supplement: Table S1 — SSR loci associated with MTKW and TKW in 5 environments by Tassel 2.1(P<0.05). (DOCX) [file pone.0029432.s002.docx]

| Table S1 SSR loci associated with MTKW and TKW in 5 environments by Tassel 2.1(P<0.05) | | | | | | | | | | | | | | | | |
| --- | --- | --- | --- | --- | --- | --- | --- | --- | --- | --- | --- | --- | --- | --- | --- | --- |
| Locus |  | | *-LOG_10_ P* | | | | | | | | | | | | | |
|  | **Chr.** | **MTKW** | | | **TKW-L02** | | **TKW-L05** | | | **TKW-L06** | | **TKW-S10** | | | **TKW-Q10** | |
| *cfa2153* | 1A | 1.32 | | 1.81 | | | 1.48 | |  | |  | | |  | | |
| *barc263* | 1A |  | |  | | |  | |  | |  | | | 1.69 | | |
| *wmc84* | 1A |  | |  | | | 3.71 | |  | |  | | |  | | |
| *wmc304* | 1A | 2.89 | | 1.74 | | |  | | 2.11 | | 2.02 | | | 3.44 | | |
| *gwm135* | 1A |  | |  | | |  | |  | |  | | | 1.65 | | |
| *cfa2135* | 1A |  | |  | | |  | |  | | 1.31 | | |  | | |
| *gwm11* | 1B | 1.40 | | 3.06 | | |  | |  | | 1.69 | | |  | | |
| *gwm403* | 1B | 1.75 | |  | | | 1.47 | |  | | 2.11 | | |  | | |
| *gwm268* | 1B | 1.53 | |  | | | 1.70 | | 2.60 | | 1.47 | | |  | | |
| *barc188* | 1B |  | |  | | | 1.34 | |  | | 1.85 | | |  | | |
| *wmc719* | 1B |  | |  | | |  | | 1.33 | |  | | |  | | |
| *wmc147* | 1D | 2.37 | | 1.69 | | |  | | 1.62 | | 2.21 | | | 1.64 | | |
| *gwm337* | 1D |  | |  | | |  | |  | |  | | | 1.47 | | |
| *gwm458* | 1D |  | |  | | |  | |  | | 1.34 | | |  | | |
| *gwm636* | 2A | 1.49 | | 1.63 | | |  | |  | |  | | |  | | |
| *gwm328* | 2A |  | | 1.45 | | |  | |  | |  | | |  | | |
| *gwm294* | 2A |  | |  | | | 1.98 | |  | |  | | |  | | |
| *gwm275* | 2A | 1.41 | | 1.44 | | | 1.42 | |  | |  | | |  | | |
| *gwm515* | 2A |  | | 1.62 | | |  | |  | | 1.45 | | |  | | |
| *gwm425* | 2A |  | |  | | | 1.95 | |  | |  | | |  | | |
| *gwm558* | 2A |  | |  | | | 1.38 | |  | |  | | |  | | |
| *gwm312* | 2A | 2.54 | | 1.54 | | | 2.33 | | 3.05 | | 2.39 | | |  | | |
| *gwm372* | 2A | 1.43 | |  | | | 1.59 | | 2.66 | |  | | |  | | |
| *barc349* | 2B |  | | 2.08 | | | 2.55 | | 1.31 | |  | | |  | | |
| *gwm148* | 2B |  | |  | | | 1.45 | |  | |  | | |  | | |
| *gwm526* | 2B |  | |  | | |  | |  | | 1.30 | | | 1.41 | | |
| *gwm501* | 2B |  | | 1.93 | | |  | |  | |  | | |  | | |
| *gwm349* | 2D |  | |  | | | 1.44 | |  | |  | | |  | | |
| *gwm539* | 2D |  | | 1.47 | | |  | |  | |  | | |  | | |
| *cfd270* | 2D |  | |  | | | 1.76 | |  | |  | | |  | | |
| *cfd161* | 2D |  | |  | | |  | |  | | 1.33 | | |  | | |
| *barc219* | 2D | 1.39 | |  | | |  | |  | |  | | | 1.92 | | |
| *cfd50* | 2D |  | |  | | |  | |  | | 1.34 | | |  | | |
| *cfe68* | 2D |  | | 1.63 | | |  | |  | |  | | |  | | |
| *barc310* | 3A |  | |  | | |  | |  | |  | | | 1.47 | | |
| *gwm218* | 3A |  | | 1.52 | | |  | | 1.45 | |  | | |  | | |
| *cfa2234* | 3A | 1.89 | |  | | |  | | 1.71 | | 1.43 | | | 1.52 | | |
| *barc314* | 3A |  | |  | | |  | |  | |  | | | 1.43 | | |
| *gwm156* | 3B | 1.44 | | 1.97 | | | 1.47 | |  | | 1.61 | | |  | | |
| *wmc334* | 3B |  | |  | | | 1.44 | |  | |  | | |  | | |
| *gwm566* | 3B |  | |  | | |  | |  | | 2.06 | | |  | | |
| *gwm284* | 3B |  | |  | | | 1.71 | |  | |  | | |  | | |
| *barc84* | 3B | 1.32 | |  | | |  | |  | |  | | | 1.99 | | |
| *gwm547* | 3B | 2.06 | | 1.42 | | |  | | 2.30 | | 1.83 | | | 1.51 | | |
| *gwm161* | 3D | 1.31 | |  | | | 2.28 | |  | |  | | |  | | |
| *cfd34* | 3D |  | |  | | |  | | 1.33 | |  | | |  | | |
| *barc42* | 3D |  | | 1.61 | | |  | |  | |  | | |  | | |
| *gwm664* | 3D |  | |  | | |  | | 1.57 | |  | | | 1.70 | | |
| *gwm383* | 3D |  | |  | | |  | | 1.38 | |  | | |  | | |
| *cfd223* | 3D |  | | 1.33 | | |  | |  | |  | | |  | | |
| *cfe300* | 4A |  | |  | | |  | |  | |  | | | 1.96 | | |
| *wmc125* | 4B |  | |  | | | 1.51 | |  | |  | | |  | | |
| *barc288* | 4D |  | |  | | |  | |  | |  | | | 1.47 | | |
| *gwm609* | 4D |  | |  | | |  | |  | | 1.55 | | |  | | |
| *wmc705* | 5A |  | |  | | |  | |  | | 1.60 | | |  | | |
| *barc117* | 5A | 1.59 | |  | | |  | |  | | 1.37 | | |  | | |
| *barc1* | 5A |  | | 2.59 | | | 1.36 | |  | |  | | |  | | |
| *barc141* | 5A |  | |  | | |  | |  | | 1.34 | | |  | | |
| *barc330* | 5A |  | |  | | | 1.32 | |  | |  | | |  | | |
| *gwm617* | 5A |  | |  | | | 1.38 | |  | |  | | |  | | |
| *barc56* | 5A | 1.62 | | 1.60 | | |  | |  | | 3.34 | | |  | | |
| *cfe186* | 5A |  | |  | | |  | |  | |  | | | 1.45 | | |
| *gwm234* | 5B | 1.75 | | 4.17 | | | 1.87 | | 1.84 | | 1.77 | | |  | | |
| *wmc415* | 5B | 1.37 | |  | | |  | |  | | 1.46 | | | 1.43 | | |
| *cfa2070* | 5B |  | |  | | |  | |  | | 1.62 | | |  | | |
| *gwm554* | 5B |  | | 1.61 | | |  | |  | |  | | |  | | |
| *gwm554* | 5B |  | |  | | |  | | 1.35 | |  | | |  | | |
| *gwm408* | 5B |  | |  | | |  | |  | |  | | | 1.52 | | |
| *gwm205* | 5D |  | |  | | | 1.40 | |  | |  | | |  | | |
| *cfd266* | 5D | 1.42 | | 1.67 | | | 1.38 | | 1.46 | |  | | |  | | |
| *gwm174* | 5D | 2.80 | | 1.72 | | |  | | 4.18 | | 2.68 | | | 2.03 | | |
| *gwm271* | 5D |  | | 1.83 | | |  | |  | |  | | |  | | |
| *gwm212* | 5D |  | |  | | |  | |  | | 1.71 | | |  | | |
| *barc322* | 5D |  | | 1.47 | | |  | |  | |  | | |  | | |
| *barc177* | 5D |  | |  | | |  | |  | |  | | | 1.82 | | |
| *gwm494* | 6A | 1.45 | |  | | |  | | 2.03 | |  | | |  | | |
| *gwm617* | 6A |  | |  | | |  | |  | | 1.79 | | | 1.56 | | |
| *gwm356* | 6A | 1.76 | |  | | |  | | 1.31 | | 1.38 | | | 1.70 | | |
| *gwm82* | 6A |  | |  | | |  | |  | |  | | | 1.31 | | |
| *gwm193* | 6B | 2.24 | |  | | |  | |  | | 2.74 | | |  | | |
| *barc24* | 6B |  | |  | | | 1.34 | |  | |  | | |  | | |
| *barc178* | 6B | 1.35 | |  | | |  | |  | |  | | |  | | |
| *barc134* | 6B |  | |  | | |  | |  | |  | | | 1.35 | | |
| *gdm127* | 6D |  | |  | | |  | |  | | 1.30 | | |  | | |
| *barc202* | 6D |  | |  | | |  | |  | | 1.71 | | |  | | |
| *gwm55* | 6D | 2.80 | | 3.08 | | | 1.92 | | 1.63 | | 4.09 | | |  | | |
| *cfd47* | 6D |  | |  | | |  | |  | |  | | | 2.89 | | |
| *cfd60* | 6D | 1.34 | |  | | |  | |  | | 1.75 | | |  | | |
| *gpw7079* | 6D |  | | 1.77 | | |  | |  | |  | | |  | | |
| *gwm471* | 7A | 1.66 | | 1.61 | | |  | | 1.61 | |  | | | 1.30 | | |
| *wmc479* | 7A |  | | 1.47 | | |  | |  | |  | | |  | | |
| *wmc168* | 7A | 1.80 | |  | | |  | | 1.70 | | 2.12 | | |  | | |
| *wmc17* | 7A | 1.98 | |  | | | 2.42 | | 1.49 | | 2.38 | | | 1.34 | | |
| *cfa2257* | 7A | 2.70 | | 3.00 | | | 2.19 | | 2.26 | | 1.78 | | | 1.60 | | |
| *wmc286* | 7A | 1.61 | |  | | |  | | 2.29 | |  | | | 1.63 | | |
| *cwm461* | 7A |  | |  | | |  | |  | |  | | | 1.38 | | |
| *gwm400* | 7B |  | |  | | | 1.90 | |  | |  | | |  | | |
| *wmc76* | 7B |  | |  | | |  | | 1.50 | |  | | |  | | |
| *barc278* | 7B |  | |  | | |  | |  | | 1.98 | | |  | | |
| *gwm146* | 7B |  | | 1.49 | | |  | |  | |  | | |  | | |
| *gwm295* | 7D |  | |  | | | 1.90 | |  | |  | | |  | | |
| *barc154* | 7D |  | | 2.23 | | |  | |  | |  | | |  | | |
| *barc92* | 7D |  | | 1.64 | | |  | |  | |  | | |  | | |
| *barc352* | 7D |  | |  | | | 1.66 | |  | |  | | |  | | |
| *gwm44* | 7D |  | |  | | |  | | 1.57 | |  | | |  | | |
| *gdm150* | 7D |  | | 2.70 | | |  | |  | | 1.69 | | |  | | |
| *wmc157* | 7D | 1.34 | |  | | |  | |  | |  | | |  | | |
| Associated loci | | **32** | | | | **35** | | **33** | | **27** | | | **39** | | | **30** |
