# Supplementary material for: Identifying Loci Influencing 1,000-Kernel Weight in Wheat by Microsatellite Screening for Evidence of Selection during Breeding
Source: PLoS One. 2012 Feb 6;7(2):e29432. doi: 10.1371/journal.pone.0029432 (PMC3273457; doi:10.1371/journal.pone.0029432)
Supplement: Table S2 — Frequency change of favored alleles at the 22 loci associated with MTKW in landraces and modern varieties. (DOCX) [file pone.0029432.s003.docx]

| Table S2 The frequency change of favored alleles at the 22 loci associated with MTKW in landraces and modern varieties | | | | | | | |
| --- | --- | --- | --- | --- | --- | --- | --- |
|  | | | | | | | |
| Loci | **Chr.** | **Genetic position (cM)** | **Favored allele (bp)** | **Freq. (%)** | | ***PIC*** | |
|  |  |  |  | **Landraces** | **Modern var.** | **Landraces** | **Modern var.** |
| *cfa2153* | 1A | 15.44 | 198 | 20.38 | 40.00 | 0.67 | 0.47 |
| *wmc304* | 1A | 52 | 126 | 7.01 | 30.48 | 0.62 | 0.69 |
| *gwm11* | 1B | 57 | 199 | 14.65 | 16.19 | 0.68 | 0.69 |
| *gwm403* | 1B | 61.44 | 134 | 22.93 | 20.95 | 0.64 | 0.57 |
| *gwm268* | 1B | 75.24 | 230 | 5.10 | 12.38 | 0.79 | 0.80 |
| *wmc147* | 1D | 0 | 150 | 16.56 | 30.48 | 0.45 | 0.40 |
| *gwm275* | 2A | 56.12 | 110 | 3.18 | 23.81 | 0.76 | 0.76 |
| *gwm312* | 2A | 79.26 | 190 | 11.46 | 23.81 | 0.52 | 0.53 |
| *gwm372* | 2A | 80.45 | 331 | 1.91 | 12.38 | 0.68 | 0.71 |
| *cfa2234* | 3A | 107.38 | 142 | 26.11 | 70.48 | 0.33 | 0.30 |
| *gwm156* | 3B | 40.05 | 311 | 3.18 | 23.81 | 0.75 | 0.74 |
| *gwm547* | 3B | 100 | null | 7.64 | 17.14 | 0.13 | 0.26 |
| *barc56* | 5A | 18.8 | 119 | 12.10 | 51.43 | 0.29 | 0.44 |
| *gwm234* | 5B | 20.57 | 237 & 239 | 5.73 | 33.33 | 0.75 | 0.79 |
| *wmc415* | 5B | 61.29 | 154 | 15.29 | 24.76 | 0.44 | 0.42 |
| *cfd266* | 5D | 22 | 167 | 4.46 | 18.10 | 0.31 | 0.49 |
| *gwm174* | 5D | 51.85 | 191 | 27.39 | 60.00 | 0.73 | 0.58 |
| *gwm55* | 6D | 83.45 | 130 | 13.38 | 34.29 | 0.72 | 0.72 |
| gwm471 | 7A | 1 | 109 | 3.82 | 9.52 | 0.82 | 0.82 |
| *wmc168* | 7A | 32.9 | 307 | 7.01 | 11.43 | 0.49 | 0.51 |
| *wmc17* | 7A | 89.17 | 182 and 184 | 19.11 | 64.76 | 0.63 | 0.64 |
| *cfa2257* | 7A | 91.89 | 129 | 6.37 | 41.90 | 0.34 | 0.40 |
|  |  |  |  |  |  |  |  |
