# Supplementary material for: Identifying Loci Influencing 1,000-Kernel Weight in Wheat by Microsatellite Screening for Evidence of Selection during Breeding
Source: PLoS One. 2012 Feb 6;7(2):e29432. doi: 10.1371/journal.pone.0029432 (PMC3273457; doi:10.1371/journal.pone.0029432)
Supplement: Table S3 — Frequency change of major alleles at the 44 loci with higher PIC than the mean (0.54) in landraces and modern varieties. (DOCX) [file pone.0029432.s004.docx]

| Table S3 Frequency change of major alleles at the 44 loci with higher *PIC* than the mean (0.54) in landraces and modern varieties | | | | | | | |
| --- | --- | --- | --- | --- | --- | --- | --- |
| Loci | **Chr.** | **Genetic position (cM)** | **Major allele in modern var. (bp)** | **Freq.(%)** | | ***PIC*** | |
|  |  |  |  |  |  |  |  |
|  |  |  |  | **Landraces** | **Modern var.** | **Landraces** | **Modern var.** |
| *Xgpw7072* | 1A | 13.00 | 213 | 39.33 | 30.69 | 0.64 | 0.68 |
|  |  |  | 222 | 36.00 | 30.69 |  |  |
| *Wmc59* | 1A | 112.88 | 215 | 16.67 | 29.17 | 0.72 | 0.75 |
|  |  |  | 219 | 38.44 | 17.19 |  |  |
| *Gwm273* | 1B | 54.54 | 192 | 20.39 | 40.50 | 0.76 | 0.73 |
| *Gwm413* | 1B | 59.55 | 90 | 15.63 | 26.67 | 0.79 | 0.77 |
| *Barc152* | 1D | 25.10 | 130 | 27.93 | 27.27 | 0.75 | 0.78 |
| *Gwm106* | 1D | 27.8 | 128 | 11.27 | 19.89 | 0.87 | 0.88 |
| *Gwm95* | 2A | 56.12 | 117 | 26.76 | 45.21 | 0.73 | 0.66 |
| *Gwm339* | 2A | 56.12 | 156 | 29.11 | 32.18 | 0.72 | 0.73 |
| *Gwm388* | 2B | 58 | 166 | 28.57 | 36.27 | 0.74 | 0.68 |
| *Gwm120* | 2B | 89.18 | 134 | 39.35 | 39.63 | 0.75 | 0.76 |
| *Gwm210* | 2D | 17.31 | 188 | 21.62 | 20.88 | 0.82 | 0.83 |
| *Wmc18* | 2D | 45.88 | 243 | 37.07 | 39.16 | 0.73 | 0.72 |
| *Barc310* | 3A | 23.40 | 196 | 44.35 | 45.83 | 0.65 | 0.64 |
| *Barc12* | 3A | 25.32 | 184 | 22.41 | 32.14 | 0.67 | 0.74 |
| *Wmc78* | 3B | 29.55 | 256 | 15.15 | 27.78 | 0.73 | 0.72 |
| *Gwm77* | 3B | 46.36 | 161 | 17.70 | 23.15 | 0.82 | 0.81 |
| *Gwm52* | 3D | 64.26 | 144 | 44.44 | 43.75 | 0.62 | 0.65 |
| *Cfd152* | 3D | 101.18 | 288 | 35.11 | 38.20 | 0.66 | 0.69 |
| *Gwm397* | 4A | 68.74 | 173 | 17.94 | 25.00 | 0.82 | 0.80 |
| *Gwm637* | 4A | 93.86 | 173 | 26.83 | 22.89 | 0.76 | 0.77 |
| *Gwm368* | 4B | 35.57 | 244 | 34.03 | 39.86 | 0.59 | 0.64 |
| *Gwm375* | 4B | 41.38 | 163 | 44.35 | 43.75 | 0.59 | 0.64 |
| *Gwm624* | 4D | 112.75 | 124 | 39.72 | 36.96 | 0.72 | 0.75 |
| *Gwm609* | 4D | 115.7 | 111 | 39.58 | 43.33 | 0.72 | 0.71 |
| *Cfd39* | 5A | 83.19 | 157 | 21.62 | 21.21 | 0.82 | 0.82 |
| *Wmc524* | 5A | 101.92 | 221 | 36.84 | 39.36 | 0.65 | 0.63 |
| *Gwm544* | 5B | 41.41 | 171 | 16.13 | 20.73 | 0.80 | 0.82 |
| *Gwm540* | 5B | 44.63 | 127 | 17.36 | 26.63 | 0.79 | 0.79 |
| *Gwm192* | 5D | 8 | 200 | 16.39 | 33.33 | 0.76 | 0.74 |
| Gwm182 | 5D | 50.98 | 158 | 29.48 | 40.32 | 0.65 | 0.61 |
|  |  |  | 160 | 39.55 | 36.02 |  |  |
| *Barc3* | 6A | 66.01 | 197 | 38.19 | 38.61 | 0.67 | 0.66 |
| *Wmc201* | 6A | 109.11 | 247 | 25.37 | 38.96 | 0.68 | 0.72 |
| *Wmc486* | 6B | 6.05 | 204 | 48.18 | 42.42 | 0.61 | 0.60 |
| *Gwm361* | 6B | 105.85 | 135 | 40.13 | 48.04 | 0.65 | 0.52 |
| *Gwm469* | 6D | 39.00 | 172 | 14.89 | 29.69 | 0.80 | 0.78 |
| *Gwm194* | 6D | 100.42 | 129 | 43.05 | 47.92 | 0.63 | 0.60 |
| *Wmc158* | 7A | 0.00 | 363 | 13.48 | 27.17 | 0.79 | 0.75 |
|  |  |  | 365 | 9.22 | 33.70 |  |  |
|  |  |  | 369 | 29.08 | 8.70 |  |  |
|  |  |  | 371 | 21.28 | 6.52 |  |  |
| Wmc479 | 7A | 29.00 | 196 | 42.86 | 28.57 | 0.64 | 0.73 |
|  |  |  | 198 | 32.14 | 28.57 |  |  |
| *Gwm333* | 7B | 46.00 | 150 | 20.14 | 27.47 | 0.69 | 0.70 |
| *Wmc276* | 7B | 108.04 | 304 | 26.83 | 24.05 | 0.77 | 0.79 |
| *Cfd31* | 7D | 45.24 | 244 | 26.00 | 33.33 | 0.77 | 0.79 |
| *Barc172* | 7D | 148.29 | 172 | 29.63 | 33.33 | 0.76 | 0.73 |
|  |  |  |  |  |  |  |  |
